# Closed-Loop Auditory Stimulation to Guide Respiration: Preliminary Study to Evaluate the Effect on Time Spent in Sleep Initiation during a Nap

**DOI:** 10.3390/s23146468

**Published:** 2023-07-17

**Authors:** Heenam Yoon, Sang Ho Choi

**Affiliations:** 1 Department of Human-Centered Artificial Intelligence, Sangmyung University, Seoul 03016, Republic of Korea; 2 School of Computer and Information Engineering, Kwangwoon University, Seoul 01897, Republic of Korea; shchoi@kw.ac.kr

**Keywords:** closed-loop system, auditory stimulation, sleep initiation, respiration, nap

## Abstract

Various stimulation systems to modulate sleep structure and function have been introduced. However, studies on the time spent in sleep initiation (TSSI) are limited. This study proposes a closed-loop auditory stimulation (CLAS) to gradually modulate respiratory rhythm linked to the autonomic nervous system (ANS) activity directly associated with sleep. CLAS is continuously updated to reflect the individual’s current respiratory frequency and pattern. Six participants took naps on different days with and without CLAS. The average values of the TSSI are 14.00 ± 4.24 and 9.67 ± 5.31 min in the control and stimulation experiments (*p* < 0.03), respectively. Further, the values of respiratory instability and heart rate variability differ significantly between the control and stimulation experiments. Based on our findings, CLAS supports the individuals to gradually modulate their respiratory rhythms to have similar characteristics observed near sleep initiation, and the changed respiratory rhythms influence ANS activities, possibly influencing sleep initiation. Our approach aims to modulate the respiratory rhythm, which can be controlled intentionally. Therefore, this method can probably be used for sleep initiation and daytime applications.

## 1. Introduction

Sleep is essential to provide intrinsic functions and to link with daytime activities. Sleep is crucial in physical and mental recovery [1,2,3], memory consolidation [4], and hormone acceleration [5]. Insufficient sleep increases daily sleepiness [6] and accident rates [7] and decreases cognitive function [8]. Polysomnography (PSG) is a standard measure to evaluate sleep [9]. PSG provides information on sleep structure and sleep-related disorders. Information associated with sleep includes total sleep time (TST), sleep efficiency (SE), sleep onset latency (SOL), wake after sleep onset (WASO), and the percentage of each sleep stage [9], which are used to evaluate sleep structure anomalies and sleep disorders. For example, SE and TST are used to validate the overall ratio between sleep and wakefulness. SOL and WASO are used to evaluate the difficulty encountered by some individuals to fall asleep and maintain sleep [10], respectively. Thus, long-term information can be used to characterize whether an individual has sleep disorders (such as insomnia) or occasional sleep difficulties (symptoms).

Extensive studies on automatic methods to monitor sleep information using PSG data and further using data measured with wearable and/or nearable devices are underway [11,12]. Recently, non-pharmacological methods to modulate sleep structure and enhance sleep function have been introduced. According to Raymann et al., skin temperature manipulation using a thermosuit decreases nocturnal awakening and shifts sleep to deeper stages in young and elderly healthy and insomniac participants [13]. Other studies introduced bed rocking with 0.25 Hz [14,15,16]. Based on these studies, gentle rocking stimulation influences changes in electroencephalogram (EEG) characteristics and sleep structure, as well as enhances memory retention. Choi et al. proposed a weak closed-loop vibrational stimulation to modulate heart rhythm during sleep [17]. They reported that the stimulation improved the depth of slow-wave sleep and memory retention.

Respiration is a physiological rhythm fundamentally determined by the autonomic nervous system (ANS) activity [18]. However, respiration can be intentionally controlled, and respiratory modulation can also influence ANS activity. Different inhalation and exhalation ratios influenced changes in heart rate variability (HRV) parameters [19], which are used to evaluate ANS activity [20]. In addition, slow-paced respiration (SPR) can influence cardiorespiratory, cardiovascular systems, and ANS activity [21,22,23], and it is used for various applications. SPR can be used to reduce blood pressure to improve hypertension; these findings can be linked with baroreflex sensitivity, which is improved by SPR training [24]. According to Laborde et al., SPR performed before or after physical exertion positively influences the adaptation to psychological stress and inhibition [25]. In addition, the performance of executive function tasks improves after SPR compared with controls [26]. Furthermore, SPR before sleep can improve objective sleep parameters, such as SOL and SE [27], as well as subjective sleep quality [28].

External stimuli can modulate the time spent (or percentage) at each sleep stage during sleep. However, studies on time spent in sleep initiation (TSSI) are limited. Sleep is closely associated with ANS activity [29,30]. Sympathetic activity is high during wakefulness and gradually reduces with increased parasympathetic activity as sleep progresses to deeper stages [30]. Alteration in ANS activity during sleep results in characteristic changes in physiological signals, including respiration. In particular, regular and reduced respiratory rhythms are observed near the sleep onset period [31]. Respiratory modulation is available by an individual’s intention before sleep; thus, it can influence changes in ANS activity. In this context, suppose external stimulation can modulate respiration to have similar characteristics observed near sleep initiation. In that case, TSSI is expected to be changed.

Therefore, we propose a closed-loop auditory stimulation (CLAS) to gradually modulate an individual’s respiratory rhythm (frequency and pattern) and investigate its effect on sleep initiation during a nap. The characteristics of nighttime sleep and daytime nap can differ, including their durations and percentage of sleep stages; however, this study focuses on the process from wakefulness to sleep. Thus, the feasibility of the proposed method is evaluated by nap studies. We hypothesized that CLAS could support individuals to modulate their respiratory rhythms similar to those near the sleep initiation period; thus, changes in respiratory rhythms can influence ANS activity and sleep initiation. To evaluate the hypothesis, we investigate the effect of CLAS on sleep initiation, respiratory rhythms, and ANS activity based on HRV analysis.

## 2. Materials and Methods

### 2.1. Closed-Loop Auditory Stimulation (CLAS)

This study proposes CLAS that enables individuals to modulate their respiratory rhythms. The CLAS is a continuous Brownian 1/*f*^2^ noise; however, it was generated to change its amplitude according to the individual respiratory frequency. The respiratory signal was filtered using a 5th-order Butterworth high-pass filter with a cutoff frequency of 0.15 Hz and a low-pass filter with a cutoff frequency of 1 Hz to determine the characteristics of CLAS. Subsequently, the signal was smoothed using the moving-window average method with a window size of 2 s. The respiratory frequency was obtained using the dominant frequency from the FFT result of the processed signal. Finally, the amplitude of the CLAS was set to vary sinusoidally with frequency, which was 5% lower than the observed current respiratory frequency. For example, suppose the individual’s current respiratory frequency was 0.5 Hz. In that case, the CLAS with an amplitude variation of 0.475 Hz was delivered to the individual. Thus, the individuals could gradually reduce their respiratory frequencies as they breathed, according to CLAS. Here, we set the minimum frequency at 0.15 Hz to avoid individuals’ breathing discomfort with an extremely low frequency.

**Figure 1:**
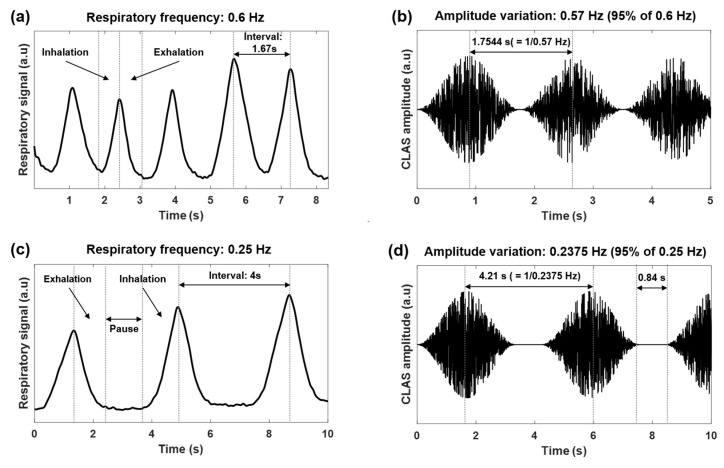
Examples of respiratory signals and CLAS generation. (**a**) An example of a respiratory signal corresponding to 0.6 Hz. Inhalation and exhalation were measured without a respiratory-pause segment. (**b**) An example of the generated CLAS when the respiratory frequency was 0.6 Hz. The amplitude of CLAS increased and decreased in intervals of 1.7544 s, determined using the inverse of 0.57 Hz (5% reduction from 0.6 Hz). The frequency was higher than 0.5 Hz; thus, the segment for amplitude maintenance was not generated. (**c**) An example of a respiratory signal with 0.25 Hz. Inhalation and exhalation were measured with the respiratory-pause segment. (**d**) An example of the generated CLAS when the respiratory frequency was 0.25 Hz. The amplitude of CLAS varied in an interval of 4.21 s, determined using the inverse of 0.2375 Hz (5% reduction from 0.25 Hz). The frequency was lower than 0.5 Hz; thus, the segment for amplitude maintenance was generated with an interval of 0.84 s, determined by 20% of 4.21 s.

The respiratory pattern differs according to respiratory frequency. When the respiratory frequency is high, continuous symmetric inhalation and exhalation are observed in the respiratory signal. In contrast, a respiratory-pause segment is observed between the exhalation and subsequent inhalation periods when the respiratory frequency is low (Figure 1). Thus, the CLAS was generated to have segments with amplitude increased, decreased, and maintained. Their durations were determined using a ratio of 4:4:2 with a stimulation frequency below 0.5 Hz. That is, suppose the amplitude variation of the CLAS was determined as 0.2 Hz. In that case, it corresponded to an interval of 5 s. Thus, the amplitude of CLAS increased, decreased for 2 s, and maintained for 1 s. Each duration could be determined separately according to the individual’s current respiratory frequency. Examples of CLAS are shown in Figure 1. The CLAS was updated approximately every 15 s. The system was developed in MATLAB R2022b (MathWorks, Natick, MA, USA).

### 2.2. Experimental Protocol and Study Participants

This study aims to evaluate the effects of CLAS on sleep initiation. We performed experiments that included daytime naps between 12:00 p.m. and 4:00 p.m. to assess the probability. Three experiments were conducted: adaptation, control, and stimulation. First, each participant visited a laboratory to adapt to an unfamiliar experimental environment and then experienced CLAS. Participants were asked to breathe comfortably (spontaneous respiration) for 15 min while lying on a bed. After a 5 min rest, they were asked to breathe following the CLAS (guided respiration). The participants were requested to stay awake with their eyes opened to prevent falling asleep. During the stimulation and control experiments, each participant took a daytime nap for approximately 1 h with and without CLAS. Each experiment was randomly ordered and conducted at least 1-week intervals. Participants were asked to avoid alcohol consumption and have sufficient sleep on the day before the experiment. In addition, caffeine intake was restricted to the day of the experiment. The study protocol was approved by the Institutional Review Board of Sangmyung University (IRB No. SMUIRB C-2022-005) and conducted in accordance with relevant guidelines and regulations.

Six subjects (2 males and 4 females, mean and standard deviation of age 23.00 ± 2.53) who satisfied the inclusion and exclusion criteria participated in this study. The inclusion criteria were (1) age between 18 and 40 years and (2) no sleep-related symptoms. The exclusion criteria were (1) a history of severe physical and psychological illness, (2) suffering from arrhythmia, (3) taking medicines that influence sleep, and (4) irregular sleep three days before the experiments. The subjects’ demographics are summarized in Table 1. All subjects provided written informed consent before participating in the study.

### 2.3. Acquisition of Physiological Signals

During the adaptation experiment, ECG and the respiratory signal from the chest belt were measured using BN-RSPEC (Biopac Systems, Inc., Goleta, CA, USA). During the stimulation and control experiments, EEG, EOG, ECG, and the respiratory signal with a chest belt were measured using MP160 (Biopac Systems, Inc., Goleta, CA, USA). A standard PSG was not conducted in this study; however, it was necessary to measure the TSSI. Thus, electrode locations were determined based on a sleep-scoring guideline from the AASM [9]. According to the guidelines, wakefulness and N1 stage were mainly determined using EEG in the occipital region and EOG. Thus, we placed electrodes to record EEG at O2-M1 locations and EOG at E1-M2 locations. All data were recorded at a sampling frequency of 500 Hz. EEG and EOG were used to automatically detect sleep initiation, ECG was used to evaluate ANS activity based on HRV analysis using R-R intervals, and the respiratory signal was used to analyze respiratory rhythms and generate CLAS.

### 2.4. Determination of Sleep Initiation

**Figure 2:**
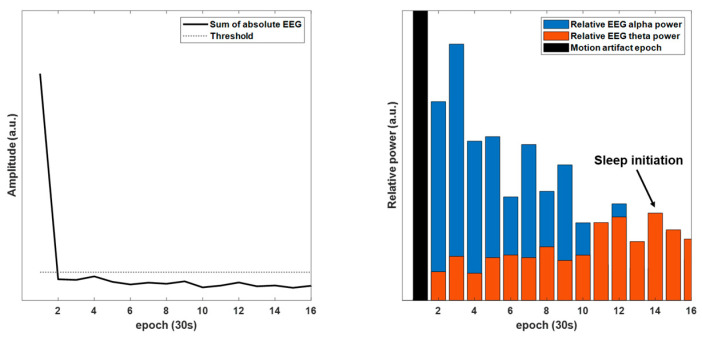
An example of sleep initiation. The epoch of sleep initiation was determined when the relative EEG theta (4–7 Hz) power was higher than the relative EEG alpha power (8–13 Hz) during two consecutive epochs without motion artifacts.

We measured EEG and EOG, which were used to score sleep stages, although a standard PSG was not performed. We developed an automatic detection method by referring to the AASM guideline to objectively determine sleep initiation [9]. According to the guideline, wakefulness is determined when more than 50% of an epoch (30 s) has an alpha rhythm (8–13 Hz) over the occipital region. In addition, wakefulness can be determined with eye blink frequency (0.5–2 Hz) and irregular rapid eye movement with high muscle tone. The N1 stage was scored if the alpha rhythm was attenuated and replaced by a low-amplitude, mixed-frequency EEG activity (4–7 Hz, known as theta rhythm) for over 50% of the epoch and slow eye movement [9]. Therefore, the epoch of sleep initiation was automatically determined as follows: (1) motion artifact-free. (2) The relative power of the theta rhythm (4–7 Hz) was higher than the relative alpha rhythm (8–13 Hz) of EEG during two consecutive epochs. The EEG signal was high-pass and low-pass filtered sequentially with 5th-order Butterworth filters at 0.1 and 30 Hz, respectively. The motion artifact indicated individual movements; thus, it could be regarded as wakefulness. The motion artifact was found as an epoch in which the sum of the absolute EEG within a time window of 30 s was higher than the threshold. In addition, using FFT, the relative powers of alpha and theta rhythms were obtained using the ratio of the power in target frequencies of EEG (alpha: 8–13 Hz and theta: 4–7 Hz) over the power of the filtered EEG within 30 s. Finally, TSSI was expressed in minutes using the detected epoch of sleep initiation. An example of this method is shown in Figure 2.

### 2.5. Respiratory Instability and HRV Analysis

The respiratory instability was evaluated to validate whether CLAS influenced the maintenance of the respiratory rhythm. The respiratory signal was high-pass and low-pass filtered with 5th-order Butterworth filters with cutoff frequencies of 0.15 Hz and 1 Hz, respectively, and subsequently processed using the moving window average method with a window size of 2 s. The dominant frequencies were obtained using data within 30 s, spectrogram analysis with 25 s overlapping, and a frequency resolution of 0.008 Hz. The respiratory instability was measured using the standard deviation of respiratory frequencies obtained over a period of 1 min in 5 s shifts.

HRV parameters [20] were calculated using the R-R intervals from the ECG. The ECG was processed using 5th-order Butterworth high-pass and low-pass filters with cutoff frequencies of 0.5 Hz and 35 Hz, respectively. R-peak locations were automatically detected and manually corrected. Time domain HRV parameters, such as the mHR and RMSSD, were calculated with R-R intervals within 30 s. In addition, frequency domain HRV parameters, such as nLF, nHF, and LFHF, were measured with R-R intervals within 5 min by 30 s shifts.

### 2.6. Statistical Evaluation

Differences in TSSI were evaluated between the control and stimulation experiments using the Wilcoxon signed-rank test. Respiratory instabilities were compared between spontaneous and guided respirations in the adaptation experiment and between respirations before sleep initiation in the control and stimulation experiments using the independent samples *t*-test. The values of HRV parameters obtained before and after sleep initiation (+15 min) were compared between the control and stimulation experiments using the independent samples *t*-test.

## 3. Results

### 3.1. Respiratory Instability in the Adaptation Experiment

**Figure 3:**
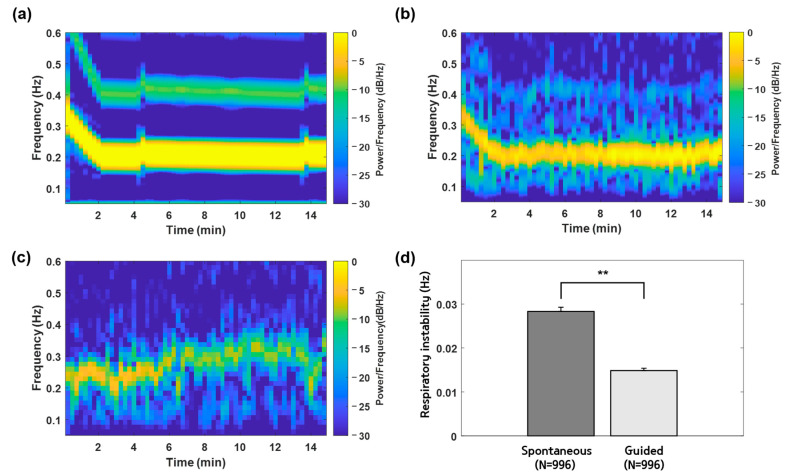
Examples of respiratory frequencies for spontaneous and guided respirations in the adaptation experiment. (**a**) An example of amplitude variation frequency of the CLAS applied to one participant. (**b**) Respiratory frequencies observed during CLAS (guided respiration) are described in (**a**). (**c**) Respiratory frequencies observed without CLAS (spontaneous respiration). (**d**) Average values of respiratory instability during spontaneous and guided respiration in the adaptation experiment for all participants. The values were presented with a mean and standard error. ** *p* < 0.001 between spontaneous and guided respiration with independent samples *t*-test. N—number of data used to obtain the values.

Examples of respiratory frequencies during spontaneous and guided respirations in the adaptation experiment are shown in Figure 3b,c. The respiratory instabilities during spontaneous and guided respirations are shown in Figure 3d. The average values of the respiratory instability were 0.0283 ± 0.0010 Hz and 0.0149 ± 0.0005 Hz (mean ± standard error) between the spontaneous and guided respirations in the adaptation experiment, respectively (*p* < 0.001, independent samples *t*-test).

### 3.2. Effect of CLAS on TSSI

**Figure 4:**
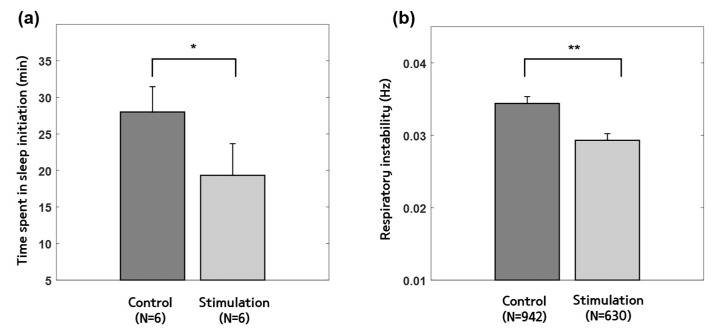
Results of (**a**) TSSI and (**b**) average values of the respiratory instability in the control and stimulation experiments. * *p* < 0.03 with Wilcoxon signed-rank test, and ** *p* < 0.001 with independent samples *t*-test, between the control and stimulation experiments. TSSI—time spent in sleep initiation, N—number of data used to obtain the values.

The results of TSSI from the control and stimulation experiments are summarized in Table 2 and Figure 4a. The average values of TSSI were 14.00 ± 4.24 min and 9.67 ± 5.31 min (mean ± standard deviation) in the control and stimulation experiments, respectively (*p* < 0.03, Wilcoxon signed-rank test). The respiratory instability is shown in Figure 4b. The average values were 0.0344 ± 0.0009 Hz and 0.0293 ± 0.0009 Hz (mean ± standard error) in the control and stimulation experiments (*p* < 0.001, independent sample *t*-test), respectively.

### 3.3. Results of HRV Analysis

The results of the HRV analysis in the control and stimulation experiments are listed in Table 3. Between the control and stimulation experiments before sleep initiation, the average values of mHR were 75.05 ± 8.69 and 73.72 ± 6.41 bpm, RMSSD were 34.84 ± 16.34 and 52.07 ± 22.06 ms, nLF were 0.61 ± 0.14 and 0.44 ± 0.12, nHF were 0.39 ± 0.14 and 0.56 ± 0.12, and LFHF were 1.86 ± 0.97 and 0.89 ± 0.44, respectively (*p* < 0.001 with independent samples *t*-test, except for mHR). In addition, between the control and stimulation experiments after sleep initiation, the average values of Mean HR were 69.81 ± 6.95 and 66.04 ± 9.11 bpm, RMSSD were 53.34 ± 19.73 and 66.76 ± 32.09 ms, nLF were 0.55 ± 0.16 and 0.47 ± 0.12, nHF were 0.45 ± 0.16 and 0.53 ± 0.12, and LFHF were 1.64 ± 1.24 and 0.98 ± 0.48, respectively (*p* < 0.001 with independent samples *t*-test).

## 4. Discussion

This study proposed CLAS and evaluated its effect on TSSI and ANS activity during naps. We observed an approximately 4.3 min decreased TSSI during the stimulation experiment compared with the control experiment (*p* < 0.03). In addition, HRV parameter values significantly differed between the stimulation and control experiments in the cases before and after sleep initiation.

We developed CLAS, in which an individual could modulate their respiratory rhythm (frequency and pattern). The designed CLAS had two characteristics: (1) the amplitude of Brownian 1/*f*^2^ noise sinusoidally increased and decreased with 95% of the current individual’s respiratory frequency. (2) The CLAS could have three phases: amplitude increased, decreased, and maintained as the observed individual’s respiratory frequency decreased (see Figure 1). Most studies used fixed stimulation frequencies to modulate an individual’s respiratory rhythm [21,22,23,25,26,27]. However, CLAS was generated based on an individual’s current respiratory rhythm; thus, we expected it would gradually change the respiration with minimal discomfort. In addition, CLAS could be adaptively updated when an individual’s respiratory rhythm instantly and frequently changed.

CLAS was aimed to reduce and stabilize the respiratory rhythm. This was based on a previous finding that reduced and stable respiratory rhythms are observed near the sleep initiation period [31]. Thus, CLAS is expected to contribute to the change in the individual’s respiratory rhythm and to maintain the rhythm to have similar characteristics observed near the sleep initiation period. Examples of respiratory frequencies with and without CLAS are shown in Figure 3b,c, respectively. In addition, the average values of the respiratory instability are shown in Figure 3d. The values were significantly lower during guided respiration than during spontaneous respiration (*p* < 0.001). Furthermore, the values of respiratory instability before sleep initiation differed significantly between the control and stimulation experiments (*p* < 0.001), as shown in Figure 4b. Therefore, CLAS can support the maintenance of a stable respiratory rhythm.

HRV measures ANS activities by quantifying the variation in R-R intervals. Various HRV parameters could be calculated using time-domain, frequency-domain, and nonlinear methods, and they explain the level of sympathetic and parasympathetic activities. Conventional studies observed HRV parameters recorded over a 24-h period. However, based on recent studies, short-term HRV (approximately 5 min) could be used to monitor the level of sympathetic and parasympathetic activities [20]. This study performed the short-term HRV analysis using data from the control and stimulation experiments, as shown in Table 3. RMSSD, nLF, nHF, and LFHF differed significantly before sleep initiation between the control and stimulation experiments (*p* < 0.001). Those parameters, including mHR, also differed significantly after sleep initiation between the control and stimulation experiments (*p* < 0.001). Increased values of RMSSD and nHF are associated with increased parasympathetic activity. In addition, increased values of nLF are related to increased levels of sympathetic activity, and LFHF explains sympathovagal balance [20]. In this study, RMSSD and nHF increased, whereas nLF and LFHF decreased both before and after sleep initiation during the stimulation compared with the control experiment. Based on these findings, respiration under CLAS could increase parasympathetic activity before sleep initiation and further influence the maintenance of increased parasympathetic activity after sleep initiation. SPR could influence changes in the cardiorespiratory system [21,32] and ANS activities [21,22,23]. We believe that respiratory modulation using CLAS could influence ANS activity, which further influences TSSI changes.

Gender and aging are associated with ANS activities [33,34]. In this study, the comparison was performed on HRV parameters between the control and stimulation to validate the effect of CLAS on ANS activities, and consistent results were observed for each participant. Future studies conducted to investigate the effect of CLAS on different gender and age groups would help in understanding the feasibility of this study for practical applications.

Although we developed an automatic method to detect sleep initiation based on EEG signals, PSG was not performed. Thus, further studies are required for detailed analysis with an exact definition of sleep initiation. In this study, a dataset was established when each participant completed all experiments with a visit to the laboratory three times at least one-week intervals. Although we analyzed the data from a small sample size of healthy participants, we observed the consistent effect of CLAS on TSSI and ANS activity from the participants. However, the results should also be evaluated using a large dataset from nighttime sleep, including patients with problems in sleep initiation, such as insomnia. Our proposed method did not require a pre-training procedure during the daytime or before going to sleep, as reported in previous studies [27,28]. Further studies are required to ascertain whether our method with long-term training would have a better effect on TSSI. Currently, various studies have developed automatic sleep-scoring methods using data from wearable or nearable devices [11,12], which can be applied to long-term sleep monitoring at home. Our proposed approach can contribute to sleep management by combining sleep monitoring technologies.

## 5. Conclusions

This study developed a CLAS that could assist individuals in modulating their respiratory rhythms. The TSSI was reduced when the individual took a nap with the stimulation, compared with that without stimulation. CLAS supported individuals to reduce and stably maintain respiratory rhythms and influenced changes in ANS activity, particularly an increase in parasympathetic activity and a decrease in sympathetic activity. Further studies are required to evaluate our research outcomes with a large dataset of participants who have problems with sleep initiation in nighttime sleep. Respiration is a physiological rhythm that can be intentionally controlled. Therefore, further studies could determine whether the proposed method could also be used for daytime activities, such as stress management and cognitive functioning.

## Figures and Tables

**Table 1:** Demographics for study participants (mean ± standard deviation).

Variables	Values	
Gender (male/female)	2/4	
Age (years)	23.00 ± 2.53	
BMI (kg/m^2^)	23.61 ± 7.53	
	Control	Stimulation
Total recording time (min)	54.83 ± 5.46	52.33 ± 1.63

BMI—body mass index. The total recording times were insignificant between the control and stimulation experiments (*p* > 0.05, with Wilcoxon signed-rank test).

**Table 2:** Results of TSSI in the control and stimulation experiments (mean ± standard deviation).

Participants	Control (min)	Stimulation (min)
1	21.5	20.0
2	14.0	7.0
3	14.5	9.0
4	8.5	7.5
5	12.5	9.5
6	13.0	5.0
Average	14.00 ± 4.24	9.67 ± 5.31 *

* *p* < 0.03, with Wilcoxon signed-rank test.

**Table 3:** Results of HRV analysis in the control and stimulation trials (Mean ± Standard deviation).

HRV Parameters	Before Sleep Initiation		After Sleep Initiation (+15 min)	
	Control	Stimulation	Control	Stimulation
mHR (bpm)	75.05 ± 8.69	73.72 ± 6.41	69.81 ± 6.95	66.04 ± 9.11 **
RMSSD (ms)	34.94 ± 16.34	52.07 ± 22.06 **	53.34 ± 19.73	66.76 ± 32.09 **
nLF (a.u.)	0.61 ± 0.14	0.44 ± 0.12 **	0.55 ± 0.16	0.47 ± 0.12 **
nHF (a.u.)	0.39 ± 0.14	0.56 ± 0.12 **	0.45 ± 0.16	0.53 ± 0.12 **
LFHF (a.u.)	1.86 ± 0.97	0.89 ± 0.44 **	1.64 ± 1.24	0.98 ± 0.48 **

** *p* < 0.001 between the control and stimulation experiments with independent samples *t*-test. HRV—heart rate variability; mHR—heart rate; bpm—beats per minute; RMSSD—square root of the mean squared differences of R-R intervals; nLF—normalized low frequency (0.04–0.15 Hz) power; nHF—normalized high frequency (0.15–0.4 Hz) power; LFHF—LF and HF ratio.

## Data Availability

Not applicable.

## Abbreviations

AASM	American Academy of Sleep Medicine
ANS	autonomic nervous activity
CLAS	closed-loop auditory stimulation
ECG	electrocardiogram
EEG	electroencephalogram
EMG	electromyogram
EOG	electrooculogram
FFT	fast Fourier transform
HRV	heart rate variability
LFHF	ratio between LF and HF
mHR	mean heart rate
nHF	normalized high-frequency (0.15–0.4 Hz) power of R-R intervals
nLF	normalized low-frequency (0.04–0.15 Hz) power of R-R intervals
N1	non-rapid eye movement sleep stage 1
PSG	Polysomnography
RMSSD	square root of the mean squared differences of R-R intervals
SE	sleep efficiency
SOL	sleep onset latency
SPR	slow-paced respiration
TSSI	time spent in sleep initiation
TST	total sleep time
WASO	wake after sleep onset
